# Lung densitometry in postmortem computed tomography - comparison across different fatal asphyxia groups

**DOI:** 10.1007/s12024-024-00892-7

**Published:** 2024-09-03

**Authors:** Søren Reinhold Jakobsen, Lars Schellerup, Lene Warner Thorup Boel, Kasper Hansen

**Affiliations:** 1 Department of Radiology, Aarhus University Hospital, Palle Juul-Jensens Boulevard 99, Aarhus, 8200 Denmark; 2 Department of Forensic Medicine, Aarhus University, Palle Juul-Jensens Boulevard 99, Health, Aarhus, 8200 Denmark

**Keywords:** PMCT, Asphyxia, Emphysema, Homicide, Lung densitometry

## Abstract

Asphyxia as a cause of death poses a diagnostic challenge in forensic medicine due to both the diversity of underlying mechanisms, and lack of specific markers. Acute emphysema or acute alveolar dilation have long been debated as potential findings in these asphyxia cases. To further explore the supplementary findings in our forensic asphyxia cases, this study applied lung densitometry to pulmonary postmortem computed tomography (PMCT) data. Twenty asphyxia cases (including hanging (*n* = 9), manual strangulation (*n* = 4), ligature strangulation (*n* = 1), smothering (*n* = 3), and choking (*n* = 3)) and 21 matched control cases were analysed using lung densitometry parameters - specifically quantification of low attenuation areas (LAA) and the 15th percentile point of lung density (Perc15). Our data revealed statistically significantly higher lung % volume falling within LAA at -950HU (*p* = 0.04) and − 910HU (*p* = 0.043) in the asphyxia cases compared to matched controls. The Perc15 values observed were trending towards a lower attenuation corresponding to a lower density in the asphyxia group, although this result was not statistically significant (*p* = 0.13). A subgroup analysis highlighted potential differences within the asphyxia categories, notably, higher Perc15 values were observed in the choking cases. In conclusion the results from the study support the existing evidence of low pulmonary density as a potential finding in asphyxia cases and demonstrate the potential of applying lung densitometry on pulmonary postmortem computed tomography data.

## Introduction

Asphyxia in a forensic context is a condition caused by an array of non-disease traumatic events and is frequently seen in medicolegal death investigations [1]. Fatal obstructive asphyxia keeps posing challenges to the forensic pathologist due to the absence of specific pathognomonic markers.

Diagnosing asphyxia based on a postmortem examination of the body alone may be possible in cases with cyanosis, petechiae, congestion, neck markings and marked laryngeal injuries or in cases of airways blocked by an object, such as food or similar. However, it may be entirely impossible in cases of airway obstruction by soft fabric, and even more so when the body is not found at a death scene that contains all the relevant objects. Accordingly, the diagnosis relies heavily on the examination of the death scene in conjunction with forensic evidence, the sum of findings on the body and exclusion of other causes of death [2].

There has been research into uncovering supplementary markers of asphyxia to aid the medicolegal investigation. Among these potential findings are acute alveolar dilation or acute emphysema, which have been suggested as possible findings in cases of asphyxia [3, 4]. Initially this was described histomorphologically by Brinkmann in the early 1980s. The finding of acute emphysema in cases of asphyxia has since both been confirmed [3–6] and refuted [7]. With the advent of postmortem computed tomography (PMCT) in forensic medicine, several studies have investigated the findings in asphyxia cases among other causes of death, and have proposed that the lung parenchyma of the asphyxia group often presents with lower attenuation (i.e., lower distribution of Hounsfield Units (HU)) or a lower transpulmonary density gradient [8–11]. The finding of acute emphysema is not clear, however, there are indications that asphyxia does cause reduced pulmonary opacity.

The detection of pulmonary emphysema with CT in the living starts with a qualitative examination of pulmonary lesions; for that, high resolution CT is of particular advantage. As alveolar tissue is destroyed and reduced in chronic emphysema over time, a correlating reduction in overall pulmonary CT density is easily explained by lung tissue reduction through the destruction of alveolar walls [12].

The PMCT investigation of lungs based on general CT density statistics differs from clinical use. In clinical use, chronic emphysema is characterized by the destruction and thus reduction of lung tissue which then entails a left shift in a CT density histogram [13, 14]. In forensic pathology, examination of fatal obstructive asphyxia, where there is an overlap of acute pulmonary distension in conjunction with poorly understood blood or fluid distribution, yields a pulmonary density distribution that appears to differ from control cases. To that end, the density value that marks the CT density below which 15% of the CT density distribution in both lungs are distributed is termed Perc15 (15th Percentile point, Pulmonary Density (PD15)) [15–17].

The aim of the present study was to investigate if lung densitometry can be used to differentiate cases of obstructive asphyxia from matched controls.

## Materials and methods

### Study population

The study was based on a retrospective analysis of autopsy reports and PMCT scans of individuals admitted by the Danish police for autopsy at the Department of Forensic Medicine at Aarhus University, over a four-year period from August 2019 to August 2023. Inclusion criteria were asphyxia caused by manual strangulation (MS), ligature strangulation (LS), smothering (SM), choking (CH) and hanging (HA). Cases with aspiration were excluded because fluid in the airways has the potential to affect radiodensity on PMCT. Furthermore, cases with position-dependent asphyxia and compression of thorax were also not included due to conceptual difficulties.

The following exclusion criteria were applied to both the case and control groups: Postmortem Interval (PMI) > 5 days, penetrating injury to the chest, prior or current lung disease, age above 80 and below 16 years, and severe putrefaction. The level of putrefaction was addressed in the autopsy report by the forensic pathologist who had graded putrefaction as “none”, “mild”, “moderate” or “severe”, based on visual inspection and findings.

Specific causes of death in the control group expected to severely affect lung density were excluded: Exsanguination, burn victims, cold exposure victims, opioid intoxication and death following hospitalization or intensive care treatment [9–11]. The control group was matched in terms of age, height, weight and PMI.

### PMCT data

All PMCT-scans were acquired using a Canon Aquilion Prime SP system (Canon Medical Systems Europe B.V., The Netherlands). Scan parameters were 120 kV and 320 mAs, 1.0 mm slice thickness, and the projections were reconstructed with a “hard” reconstruction kernel (FC08), as recommended for lung densitometry [18]. All scans were conducted with the arms of the body placed by the side of the torso and performed as part of routine pre-autopsy examination at the department. Data analysis was conducted using the “Lung Density Analysis” plugin on a Vitrea Workstation (Canon Medical Systems Europe B.V., The Netherlands).

### Lung densitometry analysis

**Fig. 1:**
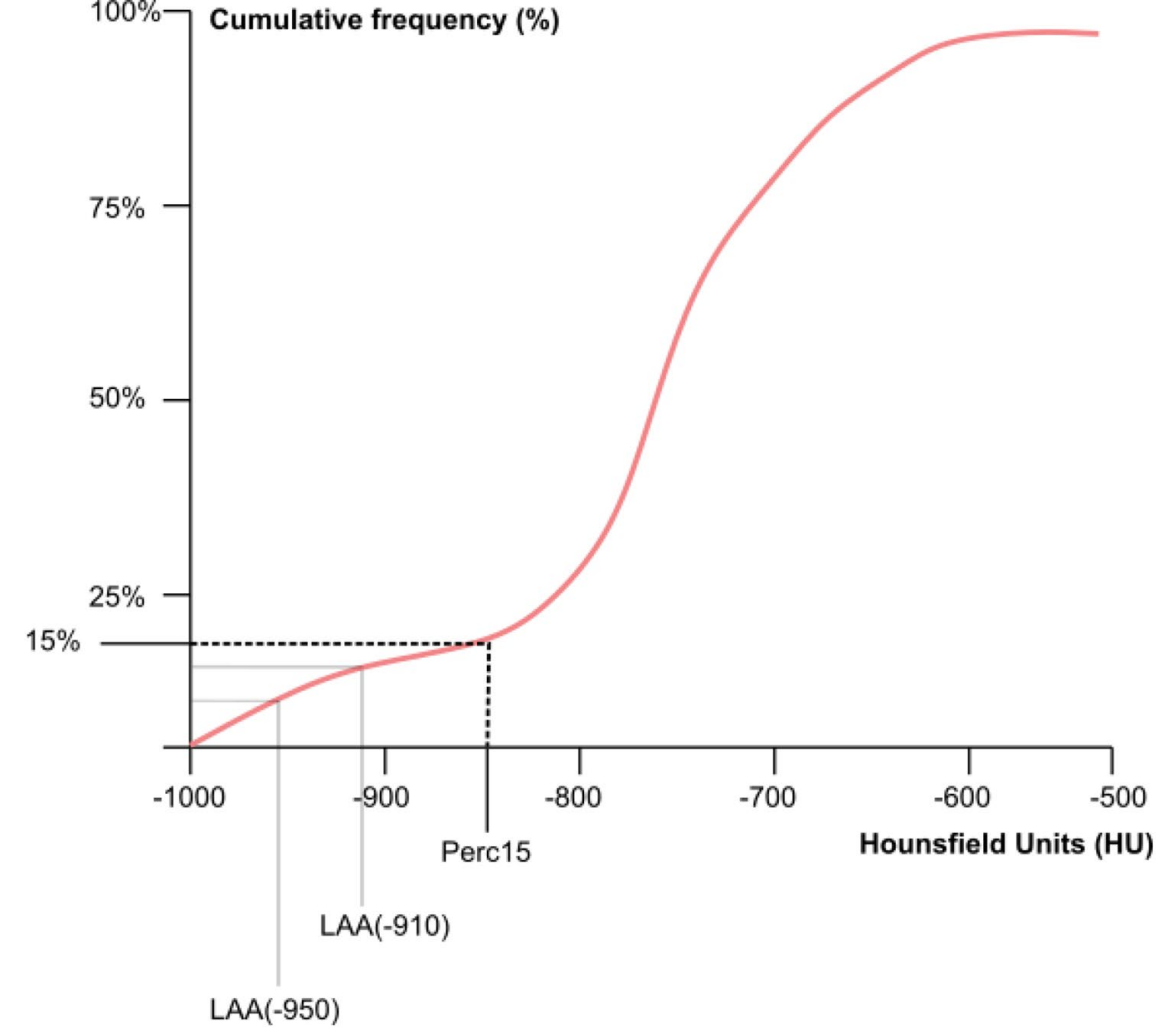
Graph illustrating the calculation of LAA and Perc15 from cumulative frequency of HU. LAA values are the percentage on the y-axis that corresponds to values of -950 HU and − 910 HU on the x-axis. Perc15 is obtained as the HU value corresponding to the 15th percentile point on the y-axis

The endpoints for this study were chosen based on previous consensus from pulmonary emphysema studies in the living [19–21]. Since there is no current agreement on the best lung densitometry endpoint, three of the most commonly applied measures were chosen. Perc15 is defined as the HU value below which 15% of the lowest density voxels are distributed. The value can be presented either as an absolute HU (Perc15) or by adding 1000 to the HU to get positive voxel-values expressed as Pulmonary Density (PD15) values in in g/L [22] (Fig. 1). For readability and coherency Perc15 was used for this study.

**Fig. 2:**
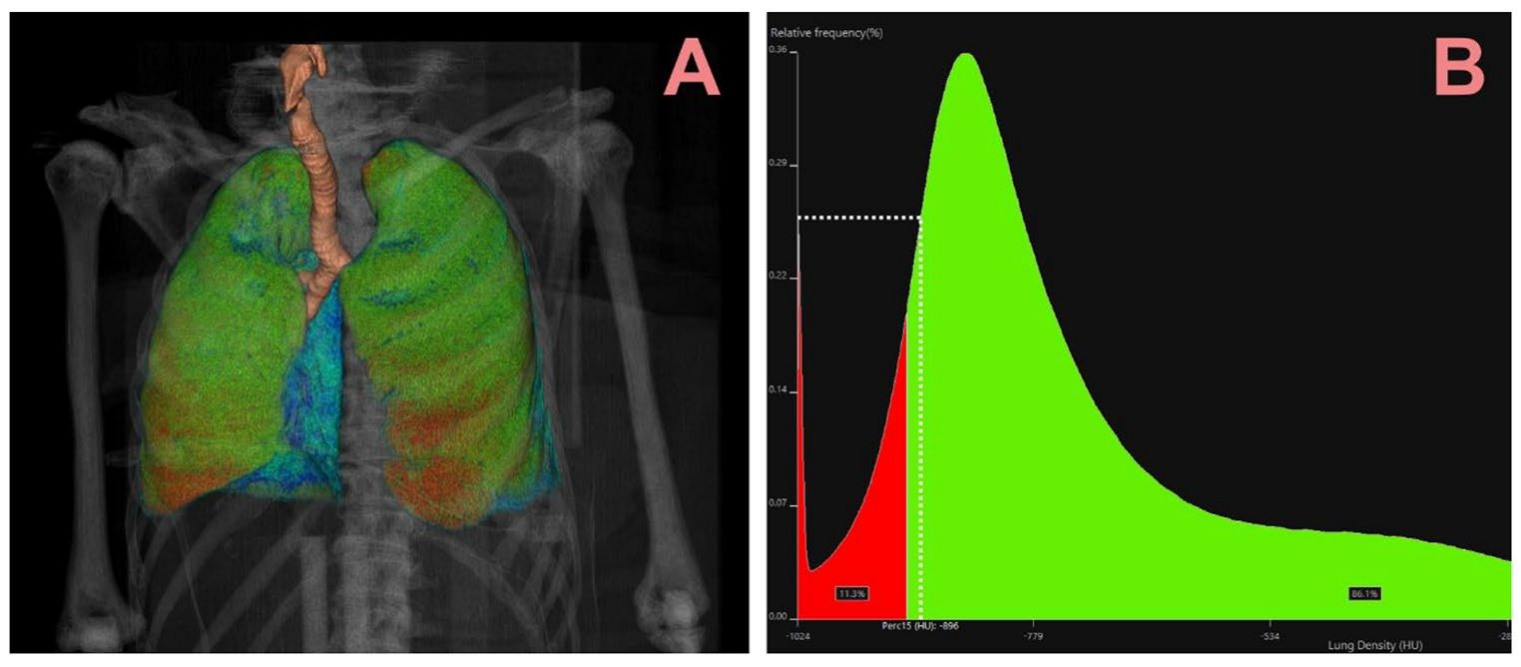
(**A**) A volume reconstruction of the thorax from an asphyxia case (hanging), with the red area illustrating low attenuation area (LAA-910). (**B**) Histogram displaying the voxel distribution of the same case (in A) displayed using the Vitrea “Lung Density Analysis” plugin

LAA is defined as the percentage of voxels in the lung with HU’s below cutoff values of either − 950 HU or -910 HU (Fig. 1). To extract the values from the PMCT-data, a semi-automatic segmentation of the lungs and airways was conducted (Fig. 2A). Each segmentation was validated and adjusted by a trained medical doctor with previous forensic and radiological experience (LS).

After segmentation both LAAs and Perc15 was obtained from voxel frequency distributions (histogram, see Fig. 2B).

### Statistical analysis

Normality was checked using visual inspection of QQ-plots and histograms. Parametric data were presented with mean and standard deviation (SD) while nonparametric data were presented with median and interquartile ranges (IQR). Statistical comparisons were conducted using Student’s t-test for parametric data and Wilcoxon Rank Sum test for nonparametric data. All statistics were performed using RStudio (RStudio Team (2020). RStudio: Integrated Development for R. RStudio, PBC, Boston, MA).

## Results

### Demographic data

Table 1 presents the demographic data, PMI, lung weight and volume for asphyxia and control cases. A total of 20 cases were included in the asphyxia group and 21 in the matched control group, respectively. The data shows a tendency towards lower body weight and longer PMI in the control group compared to the asphyxia group, but these trends were not statistically significant (*p* = 0.12 and *p* = 0.09, respectively). There were no statistically significant differences in the lung volumes extracted from segmentation data between the two groups (Table 1):

**Table 1:** Demographic data, PMI, lung weight and volume of asphyxia group and controls. Values are group means and standard deviation (SD))

	Asphyxia (*n* = 20)	Control (*n* = 21)	Statistical comparison
	Mean ± SD	Mean ± SD	
Age (years)	48.1 ± 15.2	46.9 ± 12.4	*p* = 0.79
Sex (F/M)	3/17	5/16	
Height (cm)	176.1 ± 10.4	176.0 ± 9.6	*p* = 0.99
Body weight (kg)	75.9 ± 14.2	88.1 ± 30.3	*p* = 0.12
Postmortem interval (days)	2.1 ± 1.0	2.6 ± 1.1	*p* = 0.09
Lung weight (g)	1234.5 ± 412.9	1389.0 ± 470.0	*p* = 0.27
Lung volume (mL)	2769.05 ± 952.57	2484.81 ± 865.39	*p* = 0.32

### Autopsy findings

Selected autopsy findings relevant for the asphyxia cases, including causes of death for the control group, are presented in Table 2.

**Table 2:** Autopsy findings of asphyxia and cause of death in control group

	Asphyxia						
**Subgroup**	HA (9/20)	CH (3/20)	LS (1/20)	MS (4/20)	SM (3/20)	Asphyxia (total)	Controls
**Resuscitation**	2/9	2/3	0/1	1/4	1/3	6/20	5/21
**Petechial haemorrhages**	5/9	1/3	1/1	3/4	1/3	11/20	0/21
**Fracture of cartilage or hyoid bone**	6/9	0/3	1/1	3/4	0/3	10/20	0/21
**Rib fracture**	2/9	2/3	1/1	2/4	1/3	8/20	5/21
**Emphysema (macroscopical finding)**	2/9	0/3	0/1	1/4	1/3	4/20	0/21
**Emphysema (histopathological finding)**	2/9	1/3	1/1	1/4	1/3	6/20	0/21
	**Controls**						
**Cause of death**	Cardiac	Unknown/Other	Ketone	ICH			
	8/21	6/21	4/21	3/21			

*HA: Hanging, CH: Choking, LS: Ligature Strangulation, MS: Manual Strangulation, SM: Smothering

### Lung densitometry

**Fig. 3:**
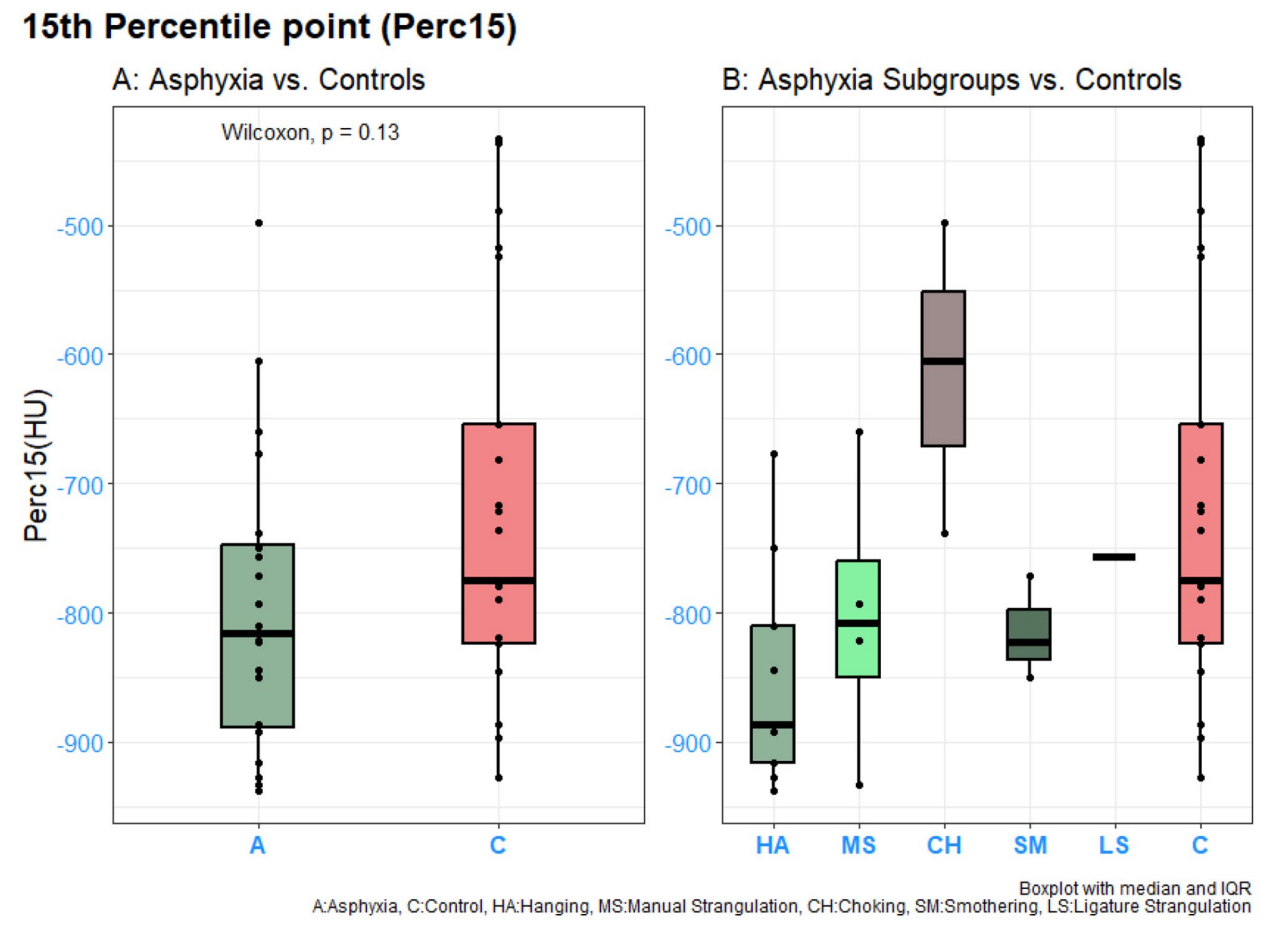
15th Percentile point (Perc15) analysis from asphyxia group (**A**) and subgroups (**B**) compared to controls

**Fig. 4:**
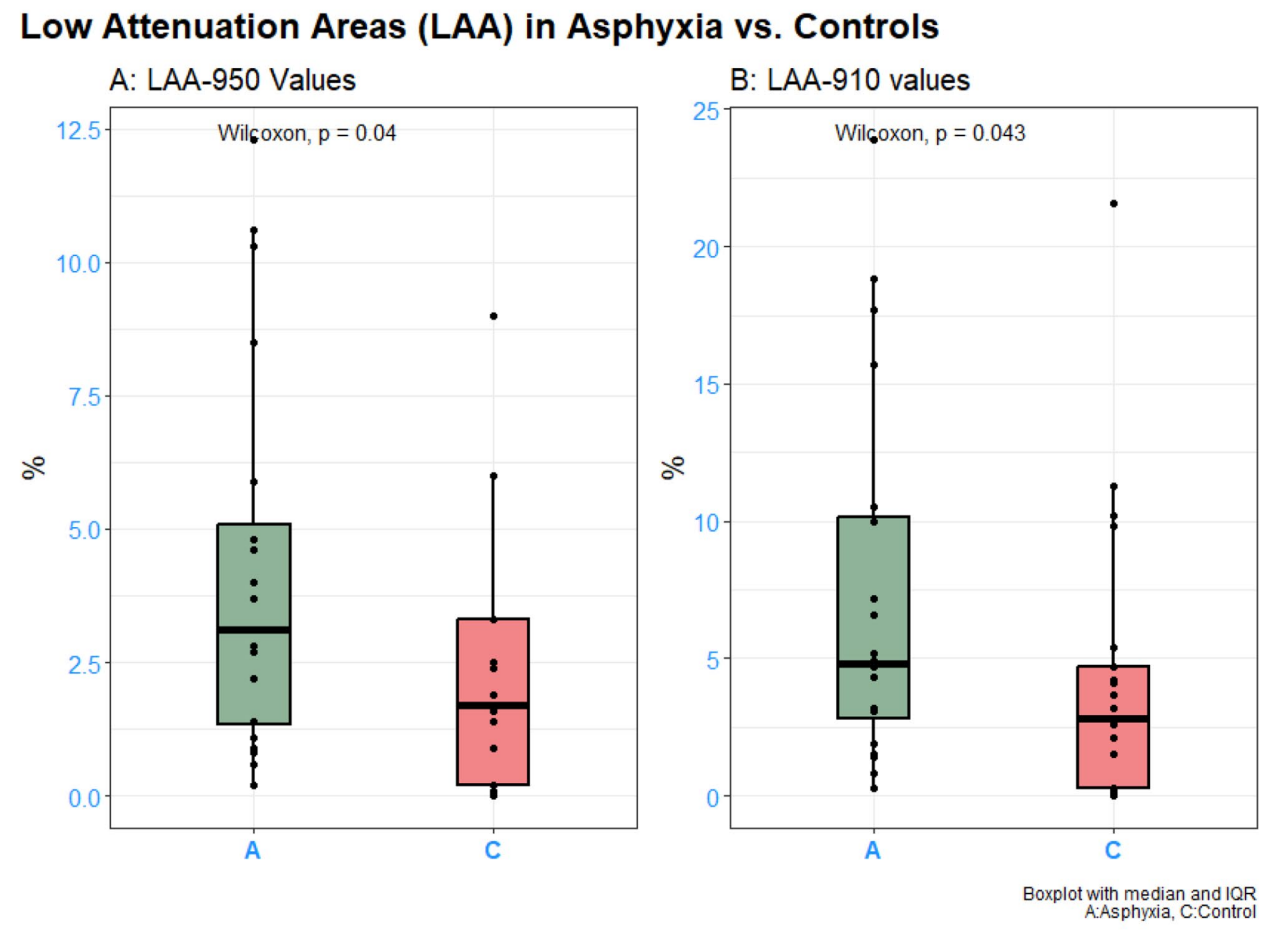
Low attenuation areas in percent with cutoff − 950HU (**A**) and − 910HU (**B**)

Results from the lung densitometry analysis are presented in Figs. 3 and 4.

No significant difference was found when comparing Perc15 values in the asphyxia groups with controls (*p* = 0.13), however, a tendency was observed where Perc15 in the asphyxia group was lower − 816 HU (IQR --886 ; -745) compared to controls − 775 HU (IQR − 886 ; -690) (Fig. 3A). In Fig. 3B, each asphyxia subgroup is presented, illustrating the high Perc15 values of the choking subgroup (-605 HU (IQR − 665 ; -555, *n* = 3)) compared to controls (-775 HU (IQR − 886 ; -690)). No statistical tests were conducted in the subgroup analysis due to the few numbers of cases available for analysis.

A greater percentage of the pulmonary voxels in asphyxia cases were of low attenuation; both LAA-950 and LAA-910 of 3.1% (IQR 1.23–4.98) and 4.8% (IQR 1.14–8.46), respectively, were significantly higher in the asphyxia group compared to the control group where LAA-950 were 1.7% (IQR 0.15–3.25) and LAA-910 of 2.8% (IQR 0.6–5).

## Discussion

The data from the present study supports existing literature stating that lower pulmonary CT density may be a significant finding in asphyxia cases [3, 4, 8, 9, 11]. In the present study lung densitometry analysis was applied to asphyxia cases and matched control cases and revealed significantly higher % volumes of low attenuation (LAA-950 and LAA-910) in the asphyxia group. The difference in Perc15 values between the asphyxia and the matched controls were not statistically significant, but the data showed a tendency towards lower values (i.e., lower density) in the asphyxia group.

When plotting subgroups of asphyxia (Fig. 3B), although data suggests that choking cases may have higher Perc15 values than controls, this finding was not statistically supported at the power provided by the limited number of cases included.

To our knowledge, no other study has reported LAA and Perc15 values on PMCT studies. Previous studies have addressed voxel distribution in relation to specific causes of death, but these were limited to means and visual inspection of voxel distribution [9, 11]. The most comparable study performed by Schweizer et al. entailed a vector analysis of the lungs in which density gradients from dependent to independent lung areas were calculated and found significantly lower density gradients in the asphyxia group compared to controls. Also, lower densities were found in some asphyxia subgroups [8] and our data supports these findings.

While lung densitometry can be used to diagnose emphysema in the living, acute emphysema in a forensic context is a different term: it is occasionally used in forensic pathology where lungs are described to appear macroscopically inflated and microscopically, alveolar walls are described to be dilated or collapsed. Histologically, findings such as fringed alveolar wall dead ends are described. At no point in forensic strangulation or asphyxia cases is a tissue reduction like chronic emphysema described or considered, nor does it seem plausible. Overall CT density differences between lungs in fatal asphyxia cases and control cases thus may have to find a different explanation than that of acute alveolar distension. Moreover, the fluid attenuation in the context of strangulation relates to the amount and distribution of blood [12].

A higher lung density of the choking group, as visually assessed from (Fig. 3B) may be due to several causes. A recent case report presented suicidal inhalation of food in the trachea, showing Negative Pressure Pulmonary Edema (NPPE) with extensive ground glass opacities (GGOs) and pulmonary edema but a lack of transpulmonary hypostatic density gradient [23]. Another theory could be aspiration of gastric content with sufficient time to develop a detectable tissue response; however, aspiration cases were excluded in this study. The pathophysiology behind these results awaits further research.

The quantitative CT analysis used in this study was relatively easy to apply and consistently reproducible [14], but did not provide a reliable methodology for identifying asphyxia cases.

Our study had certain limitations. First, relatively few asphyxia cases were included (*n* = 21), and stronger conclusions could arise using a larger study group with higher statistical power. Our inclusion period was limited due to a change of CT-scanner brand at our department in 2019. We decided not to include scans from our old system, as direct densitometry comparisons between the two systems could cause inconsistencies. Manual correction of segmentation may also have affected the result subjectively, however, as lung tissue is a high contrast organ compared to other soft tissues on PMCT, we believe such error would be negligible. Finally, it is important to note that inter-scanner variability and scan protocol setup may have affected the results, thus the absolute values should be interpreted with some caution [24] and only CT-setups- with a solid quality control procedure should be used.

Overall, our study indicates a potential for applying lung densitometry in the deceased, but further studies are needed, especially to identify reliable threshold cut-off values and subgroups of asphyxia.

## Conclusion

This study supports existing evidence that a higher percentage of very low pulmonary CT density areas can be a finding in certain asphyxia cases, however, the diverse patho-physiological mechanisms of the asphyxia subgroups may obscure these results. The study results indicate that LAA and Perc15 could be potential markers for asphyxia in PMCT, however, further research is needed to confirm these findings in a larger dataset and thus potentially find better overall threshold cut-off values for determining asphyxia in the deceased.

### Key points

1. Relevant postmortem findings in asphyxia cases have been widely debated.
2. This study investigated whether clinical emphysema diagnostics could be applied to PMCT.
3. Asphyxia deaths were associated with a greater % pulmonary voxels being of low CT attenuation compared with controls (Low attenuation areas (LAA) of -950 HU and − 910 HU).
4. PMCT lung densitometry might aid as a potential tool for identifying asphyxia in postmortem investigations.
